# Genome Sequence and Characteristics of Cluster C1 Mycobacterium smegmatis Phage EasyJones

**DOI:** 10.1128/MRA.00997-21

**Published:** 2021-11-24

**Authors:** Isabel Amaya, Duyen Bui, Ariel Egbunine, Ember Mushrush, Maggie Viland, Deborah Jacobs-Sera, Danielle Heller, Viknesh Sivanathan

**Affiliations:** a Department of Science Education, Howard Hughes Medical Institute, Chevy Chase, Maryland, USA; b Department of Biology, University of Maryland Baltimore County, Baltimore, Maryland, USA; c Department of Biological Sciences, University of Pittsburgh, Pittsburgh, Pennsylvania, USA; Portland State University

## Abstract

Bacteriophage EasyJones is a myovirus infecting Mycobacterium smegmatis mc^2^155, with a genome length and gene content similar to those of phages grouped in subcluster C1. Interestingly, EasyJones contains a gene found in a subset of C1 genomes that is similar to the well-characterized immunity repressor of subcluster A1 mycobacteriophage Bxb1.

## ANNOUNCEMENT

**FIG 1:**
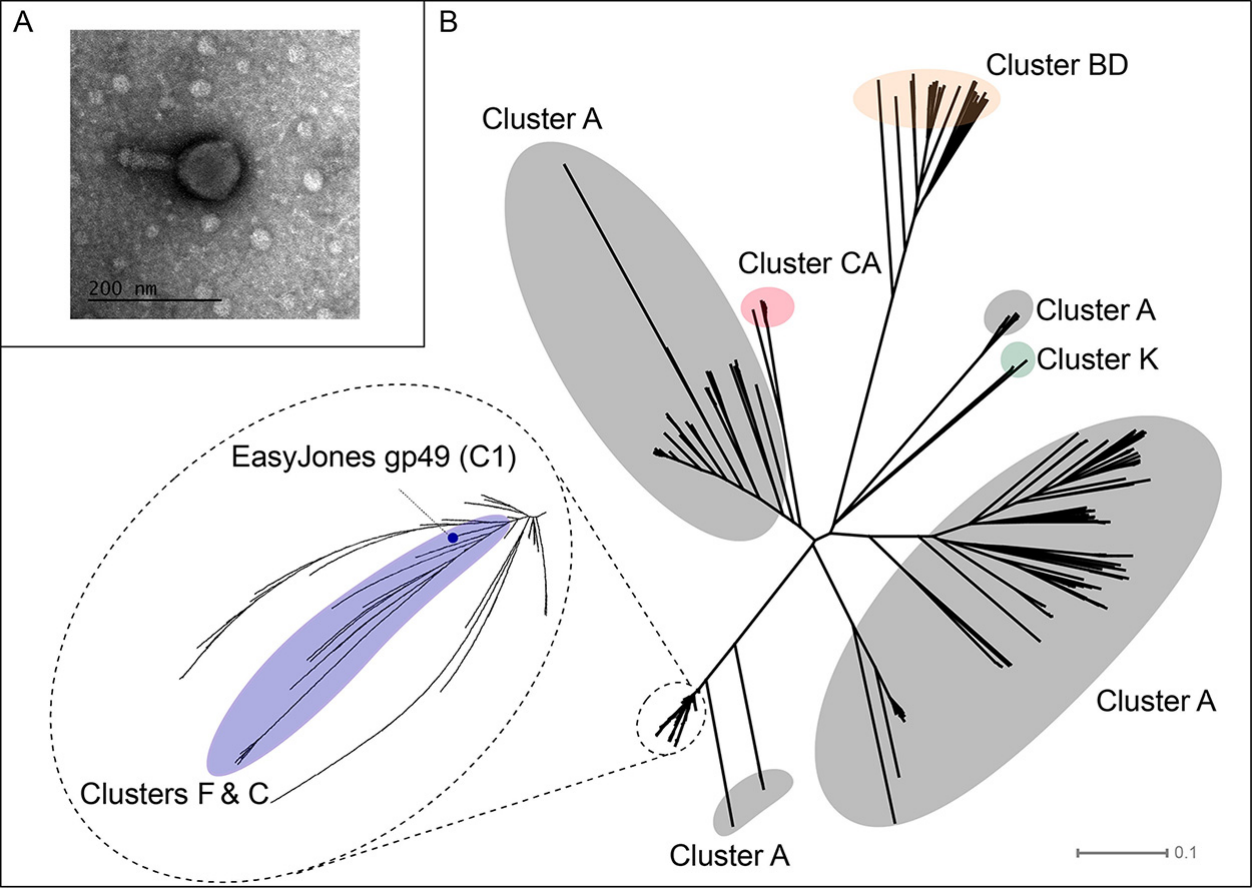
(A) Negative-stain transmission electron micrograph of EasyJones. (B) Phylogeny of EasyJones gp49 homologues. Protein sequences for 815 EasyJones gp49 homologues were aligned with Clustal Omega, and a SplitsTree network phylogeny was generated. Clades are shaded according to cluster. The position of EasyJones gp49 (blue circle) within a clade consisting of homologues from clusters C and F is indicated.

Bacteriophages are increasingly being considered as therapeutic agents for multidrug-resistant bacterial infections. Recently, several bacteriophages isolated on nonpathogenic Mycobacterium smegmatis cells were used to treat a patient with a disseminated Mycobacterium abscessus infection (1). Here, we report on EasyJones, a mycobacteriophage that was isolated, using standard methods, from soil collected from a flower bed at the University of Maryland Baltimore County (Baltimore, MD) (2). Briefly, EasyJones was extracted by washing the soil with 7H9 liquid medium, enriched in a filtered (0.2-μm pore size) wash, and purified with multiple rounds of plating on M. smegmatis mc^2^155 cells at 37°C. Top agar overlay of EasyJones results in clear plaques with a diameter of ∼0.5 mm after 24 h at 37°C. Negative-stain transmission electron microscopy revealed EasyJones to be a myovirus with a contracted tail and an isometric capsid measuring ∼88 nm in both length and diameter (Fig. 1A).

Double-stranded DNA was isolated from EasyJones using the Promega Wizard DNA cleanup kit, prepared for sequencing using the NEBNext Ultra II FS kit, and sequenced using an Illumina MiSeq sequencer to yield ∼238,000 single-end 150-bp reads, which constituted ∼231-fold coverage of the genome. Untrimmed reads were assembled and then checked for completeness using Newbler v2.9 and Consed v29, respectively, as described previously (3), resulting in a circularly permuted genome 154,315 bp in length, with a G+C content (64.7%) like that of the host bacterium (67.4%). EasyJones was assigned to phage subcluster C1 based on nucleotide similarity to members of this subcluster, using the PhagesDB database (4) and previously described criteria (5). The genome was annotated using DNA Master v5.23.6 (http://cobamide2.bio.pitt.edu), Glimmer v3.02 (6), GeneMark v3.25 (7), BLAST (8), HHpred (9), ARAGON (10), and tRNAscan-SE (11), all using default parameters. The resulting annotation process revealed a total of 267 protein-coding genes, 34 tRNAs, and 1 transfer-messenger RNA. Fifty of the protein-coding genes could be assigned functions, including the lysin A, lysin B, and holin genes.

Although a gene content similarity (GCS) comparison, performed using the PhagesDB GCS tool (4), revealed that EasyJones exhibits >83% GCS to members of subcluster C1, it adds to a small but growing list of C1 phages (15/152 phages) that possess a genomic segment encoding several additional gene products (EasyJones gp48 to gp51), including a homologue (gp49) of the well-characterized immunity repressor of subcluster A1 mycobacteriophage Bxb1 (12–14). Previous reports indicated that this genomic segment was likely acquired horizontally and that the repressor homologue does not function as a canonical immunity repressor in C1 phages (12, 14). This is consistent with the clear plaque morphology of EasyJones and the absence of any recognizable integration or partitioning systems needed to support lysogeny. Instead, this acquired repressor is thought to be maintained because it defends infected host cells from superinfection by cluster A and related phages (12, 14). A phylogenetic analysis of all 815 homologues from across clusters A, BD, C, CA, F, J, and K reveals EasyJones gp49 to be most closely related to homologues from cluster F phages, some of which also encode homologues of EasyJones gp50 and gp51 (Fig. 1B).

### Data availability.

The sequencing data for EasyJones is available in Sequence Read Archive (SRA) with accession no. SRX12475160. The genome sequence of EasyJones is available in GenBank with accession no. MZ856343.
